# The Newfound Opportunities of Wearable Systems Based on Biofeedback in the Prevention of Falls. Comment on Tanwar et al. Pathway of Trends and Technologies in Fall Detection: A Systematic Review. *Healthcare* 2022, *10*, 172

**DOI:** 10.3390/healthcare10050940

**Published:** 2022-05-19

**Authors:** Giovanni Morone, Giovanni Maccioni, Daniele Giansanti

**Affiliations:** 1 Department of Life, Health and Environmental Sciences, University of L’Aquila, 67100 L’Aquila, Italy; giovanni.morone@univaq.it; 2 Centre Tisp, The Italian National Institute of Health, 00161 Rome, Italy; gvnnmaccioni@gmail.com

We are writing to you as the corresponding authors of the interesting *systematic review* study “Pathway of Trends and Technologies in Fall Detection: A Systematic Review” [1].

We found this work to be particularly stimulating, and feel it provides great added value in the field.

Specifically, we believe, first of all, that this review has the great merit of simultaneously focusing both on important key aspects of the integration of systems for fall detection/prediction and prevention in the *health domain*, and on aspects relating to technological innovation and deployment in the three most important fields, where neuromotor problems due to pathologies or aging have a strong impact: falls from bed, falls from sitting, and falls from walking and standing. When the Special Issue “Cybersecurity and the Digital Health: An Investigation on the State of the Art and the Position of the Actors” (https://www.mdpi.com/journal/healthcare/special_issues/cybersecurity_digital_health (accessed on 1 May 2022)) [2] was launched, one of the objectives [3] was to give scholars the opportunity to broaden the boundaries of studies in this area.

Mainly, studies on cybersecurity turn more toward IT aspects, which is defined as the activity carried out in defending computers, servers, mobile devices, electronic systems, networks, and data from malicious attacks or software defaults. Therefore, what is often addressed is so-called information security and data security.

We very much appreciated your contribution because it has precisely achieved the goal of expanding and exploring new areas in this sector, *enlarging the concept of cyber-systems as tools for the development of physical security approaches for people*.

We found your work particularly interesting, wide-ranging, attractive and full of stimuli for future research. We agree with the findings of the study, that falling is one of the most serious health risks throughout the world for elderly people and for people affected by particular diseases or after recovery from accidents. In the event of a fall, as you have highlighted, considerable expenses are unfortunately necessary for patient management in the health domain. The cyber-systems developed in the field of fall risk, detection/prediction and prevention have the potential to minimize these problems. This is why we believe that your work, that has reviewed papers systematically (publications, projects, and patents), is strategic in this perspective.

As we wrote above, your study, being a review, is also very stimulating toward research initiatives to be undertaken in the future.

We would therefore like to share a reflection on this with you and with the other scholars involved in this field of research.

In the past, there has been much discussion about biofeedback systems for the prevention of falls through training and/or the use of wearable systems. Several studies have been developed based on audio, video, and vibrotactile biofeedback systems [4,5,6,7,8,9,10,11,12,13,14,15,16,17,18,19], some involving some of us as authors [8,13]. Many of these wearable systems were considered even before the smartphone boom as we know it today [9,10,11,12,13,14,15,16,17,18,19]. Some recent studies are continuing in this direction [20,21,22,23]. The use of inertial sensors, such as accelerometers, for stability control [24,25,26] integrated in wearable systems equipped with biofeedback [4,5,6,7,8,9,10,11,12,13,14,15,16,17,18,19,20,21,22,23] will certainly provide an increasingly important response in the prevention of falls.

In conclusion, we believe that your review has been a great stimulus for the Special Issue and for the scholars in general regarding future developments. Among these future developments, we also consider important those connected to wearable systems equipped with biofeedback for the prevention of falls.
